# Comparative analyses of muscle MRI and muscular function in anti-synthetase syndrome patients and matched controls: a cross-sectional study

**DOI:** 10.1186/s13075-017-1219-y

**Published:** 2017-01-25

**Authors:** Helena Andersson, Eva Kirkhus, Torhild Garen, Ragnhild Walle-Hansen, Else Merckoll, Øyvind Molberg

**Affiliations:** 1 Institute of Clinical Medicine, Department of Rheumatology, Oslo University Hospital, Pb 4950 Nydalen, 0424 Oslo, Norway; 2 Department of Radiology, Oslo University Hospital, Oslo, Norway; 3 Department of Rheumatology, Oslo University Hospital, Oslo, Norway; 4 Department of Orthopedic Surgery, Oslo University Hospital, Oslo, Norway

**Keywords:** Anti-synthetase antibody, Anti-synthetase syndrome, Myositis, Muscle magnetic resonance imaging, Muscle function

## Abstract

**Background:**

Magnetic resonance imaging (MRI) of thigh muscles is increasingly used to assess disease activity and damage extent in chronic myositis, but the validity of the findings is not clear. Here, the primary aim was to compare thigh MRI findings in patients having chronic myositis associated with anti-synthetase syndrome (ASS) and in matched healthy controls.

**Methods:**

Cross-sectional analyses of thigh muscle MRI, muscular function and creatinine kinase (CK) were performed in 68 ASS patients (median disease duration 71 months) and 67 controls matched for age and gender. MRI changes associated with disease activity (edema in muscles and fascia) and damage (fatty replacement and muscle volume reduction) were assessed semiquantitatively, giving a total MRI score of 0–78 (total edema 0–42 and total damage 0–36).

**Results:**

ASS patients had higher total MRI score than the matched controls (14.1 versus 3.0; *p* < 0.001) and less muscle strength (*p* < 0.001). Muscle edema was more frequent in ASS patients than controls (38% versus 12%), as was fatty replacement (42% versus 4%). In ASS patients, we found that the total edema score correlated with CK, but 23% of the patients with normal CK had score > 18. Muscle compartment analyses in ASS patients showed that muscle edema was most pronounced anteriorly, while fatty replacement dominated posteriorly.

**Conclusions:**

This study showed, for the first time, the magnitude of difference in muscle MRI findings between chronic myositis cases and matched controls. In ASS patients, muscle MRI appeared to provide useful complementary information to muscle strength and CK levels in the assessment of myositis.

**Electronic supplementary material:**

The online version of this article (doi:10.1186/s13075-017-1219-y) contains supplementary material, which is available to authorized users.

## Background

Anti-synthetase syndrome (ASS), first described by Marguerie et al. in 1990 [1], includes an anti-aminoacyl t-RNA synthetase (aaRS) antibody, myositis, interstitial lung disease (ILD), arthritis, Raynaud syndrome, mechanic hands and fever. Eight aaRS antibodies have been detected to date, the most common being anti-Jo1. Several sets of ASS classification criteria have been proposed, most of them with ILD and myositis as major disease items [2–4].

The impact of muscle involvement in ASS appears to differ between anti- aaRS subtypes, with myositis being most prevalent and pronounced in anti-Jo-1-positive ASS [5, 6]. Overall, the reported frequencies of myositis in ASS range from 43 to 62% [6, 7], where the incidence increases with disease duration [8]. In Jo-1-positive ASS, polymyositis (PM) was reported as the most frequent type of myositis, while dermatomyositis (DM), including amyopathic or hypomyopathic DM, seems to predominate in anti-Jo1-negative subsets [7].

Magnetic resonance imaging (MRI) of the muscles, especially the thighs, is commonly used for clinical purposes in myositis [9, 10]. As a diagnostic tool, MRI seems to be useful to differentiate between DM, PM, inclusion body myositis and non-inflammatory myopathies [11–15]. Furthermore, because the inflammatory changes in myositis may be patchy, MRI is useful to detect the best possible location for muscle biopsy [16, 17]. Research on the clinical utility of muscle MRI is expanding and recent studies have indicated that it could be useful for assessing disease activity in chronic myositis and possibly also treatment responses [9, 18, 19].

The myositis component in ASS was described recently as a distinct histological entity [20–22]. However, as far as we know, there is no study investigating whether the acute and chronic stages of ASS are marked by a specific muscle MRI pattern. Importantly, because there are no benchmark studies available on the frequency and magnitude of muscle MRI “abnormalities” in healthy populations, it has been difficult to determine the validity of this imaging modality in acute and chronic myositis. Hence, the aims for this study were: to describe and quantify muscle MRI changes in a well-defined cohort of ASS patients, most of them with established, chronic myositis, and in a gender and age-matched cohort of healthy individuals; to evaluate possible correlations between muscle MRI changes, muscle enzyme levels and muscle strength in ASS patients and controls; and to evaluate muscle outcome and possible muscle MRI patterns in ASS and its subsets with different anti-synthetase antibodies.

## Methods

### Definition of ASS and study population

The study cohort has been described elsewhere [23]. Briefly, the cohort included 68 ASS patients and 67 healthy age and sex-matched controls examined at Oslo University Hospital between September 2011 and June 2014. All examinations were done concomitantly for each participant. The ASS cohort was largely unselected, and included all of the aaRS-positive myositis cases identified in a recent population-based myositis study from south-east Norway [24]. ASS was defined as follows: a positive test of an anti-aaRS antibody; ILD according to the American Thoracic Society [25]; and/or probable/definite PM/DM according to the Bohan and Peter criteria [26].

### Serum antibody assays

Serum anti-histidyl tRNA synthetase (Jo-1) and anti-SSA autoantibodies were detected by automated ELISA (EliA; Phadia, Freiburg, Germany). Anti-threonyl (PL-7), anti-alanyl (PL-12), anti-glycyl (EJ) and anti-isoleucyl (OJ) tRNA synthetases and anti-Ro52 antibodies were detected by line blot assay (Euroline Myositis kit; Euroimmune Laboratory, Luebeck, Germany).

### Muscle MRI of the thighs

All MRI scans were obtained on 1.5-T units (Siemens, Erlangen, Germany) with coronal and axial T1-weighted spin echo and short T1 inversion recovery (STIR) sequences of the thigh muscles. Participants were asked not to exercise 24 hours before the examination. The muscles were evaluated in the anterior, posterior and medial compartments and included muscles involved in hip flexion/abduction and knee flexion/extension. Two musculoskeletal radiologists (EK, EM), unaware of the participant’s status, analyzed the MRI scans together. If any disagreement occurred, consensus was reached by discussion. Muscle edema in each compartment was evaluated in terms of extent and signal intensity, both variables using a grading scale (0–3). For the extent of edema, grade 0 was defined as no edema, grade 1 as <33.3% (minor extent), grade 2 as >33.3% to <66.6% (moderate extent) and grade 3 as >66.6% (major extent). The signal intensity of the edema was graded as 0 = no signal, 1 = low, 2 = moderate and 3 = high signal intensity. With this method, the maximum muscle edema score for each patient was 36 (18 for each thigh). Fatty replacement was scored according to the grading system suggested by Gouttalier et al. [27]; 0 = no fat, 1 = fatty streaks, 2 = muscle greater to fat, 3 = muscle equal to fat, 4 = muscle less to fat and 5 = muscle totally replaced by fat (i.e., maximum possible score 30). A Gouttalier score ≥ 2 in at least one compartment was considered pathological. Fascial edema was defined as an abnormal thick deep fascial layer with increased STIR signal, scored as present (1) or not present (0-as was muscle volume reduction. The latter was only noted when there were obvious findings: asymmetry and/or disproportionate muscle compartments. Two arbitrary scores were derived from this assessment: (A) a total edema score with a maximum score of 42 consisting of three components—edema extent (18), edema intensity (18) and presence of fascial edema (6); and (B) a total damage score (maximum score 36) including fatty replacement (30) and presence of muscle volume reduction (6). Adding A and B gave a total MRI score of 42 + 36 = 78.

### Evaluation of muscle strength and endurance

Muscle strength and muscle endurance were evaluated by two experienced physiotherapists. For muscle strength, the manual muscle test (MMT) ad modum Kendall et al. [28] was used—tested on 14 muscles (MMT14, maximum score 140) on the participants’ dominant side. The MMT14 included neck flexion/extension and peripheral and proximal muscle groups in the upper and lower extremities. For direct comparison with the MRI thigh scans, we derived a MMT4 score (maximum score 40) which included four muscles involved in hip flexion/abduction and knee flexion/extension. A slightly modified Functional Index test (FI2) [29] was used to evaluate muscle endurance, using 30 instead of 60 repetitions of neck flexion (maximum score 330).

### Muscle enzymes

Plasma creatinine kinase levels (P-CK) were measured in units per liter and were considered pathological if >2 times upper limit of normal (2ULN). For the ASS patients, CK values at the time of diagnosis were collected retrospectively from medical records.

### Statistical analysis

IBM SPSS, version 22.0 was used for statistical analyses. Normal distributed variables’ descriptive data were presented as mean and standard deviation (SD) and non-normal distributed variables were presented as median with range. Differences in continuous variables were evaluated with Student’s *t* test (using paired samples T-test) for normally distributed data, and with Wilcoxon and Mann–Whitney *U* tests for non-normally distributed data. Associations between categorical variables were detected by the chi-square test. Correlations between MRI scores, CK values, MMT scores and FI2 scores were evaluated by Pearson correlation, and between MRI scores and subgroups of ASS by Spearman rank correlation. Multiple linear regression analyses were used to evaluate possible associations between clinical variables and MRI muscle outcome.

## Results

### Patient characteristics

The ASS study cohort included 45 women and 23 men (53 with anti-Jo1, six with PL-7 and nine with PL-12 antibodies) with mean (SD) age of 47 years (13.8) at diagnosis. Median disease duration was 71 months (range 6–362). Altogether, 54 of the 68 patients (79%) had been diagnosed with myositis by their treating physician prior to study inclusion; 27 with PM, 25 with DM and two with hypomyopathic/amyopathic DM. For comparison, 97% were diagnosed with ILD [23]. The 54 ASS patients with a prior myositis diagnosis had longer median disease duration (76 months, range 6–232) than the 14 cases without myositis (22 months, range 7–230; *p* < 0.001). ASS patients positive for anti-Jo1 had myositis more often than the Jo1-negative patients (*p* < 0.001; Additional file 1: Figure S1). At study inclusion, 52/68 patients (75%) were on at least one disease-modifying drug (± oral steroids), 10 patients were on prednisolone as monotherapy and six patients did not use any immune modulating treatment.

### Muscle MRI findings in ASS versus age and sex-matched controls

**Fig. 1:**
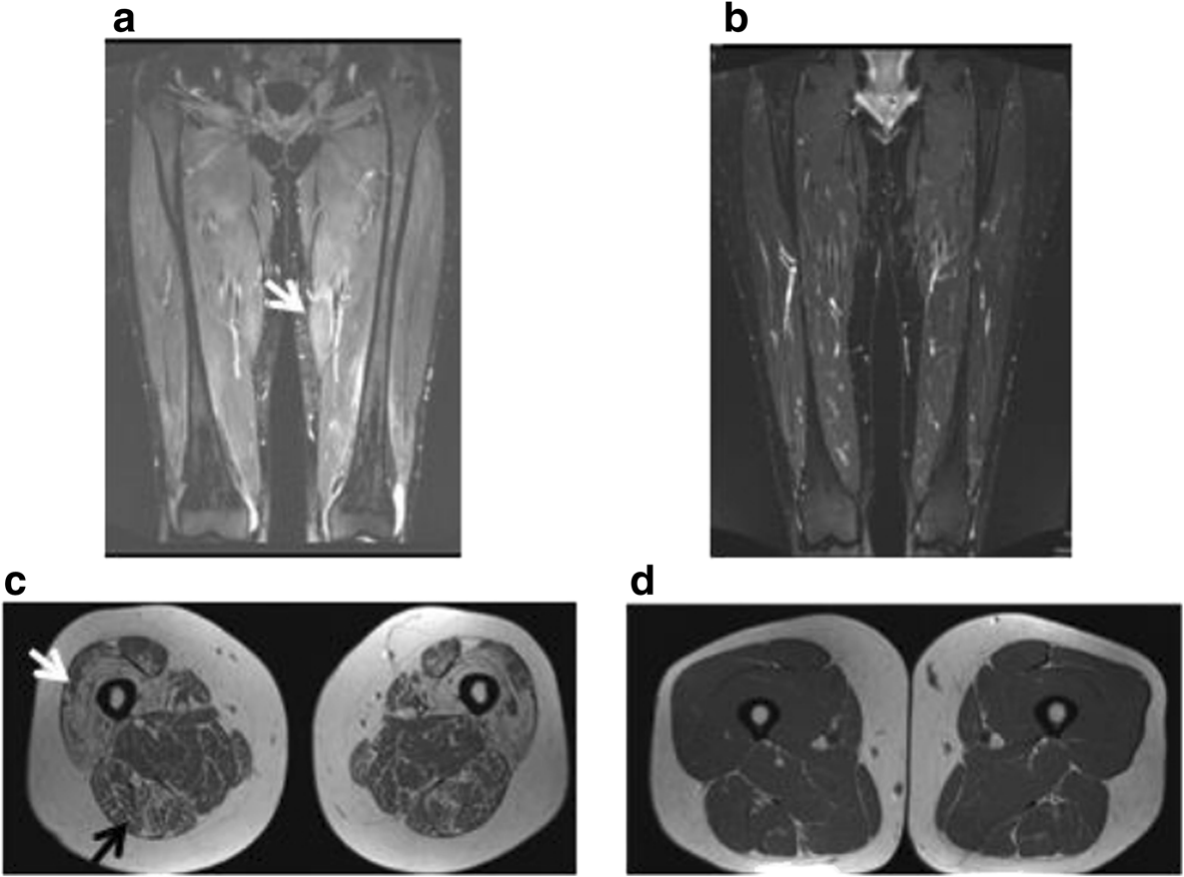
**a** Muscle MRI with coronary STIR sequence of the thighs in a patient with ASS. Compared with surrounding tissue, the muscles are generally brighter in color, most evident in the medial compartment (*arrow*), indicating general edema. The patient was scored with 36/36 points in the muscle edema score. **b** The correspondent control to the patient in (**a**) with less color difference between muscles and surrounding tissue indicating no muscle edema (score 0). **c** Axial T1-weighted image in an ASS patient with almost total fatty replacement anteriorly (Gouttalier score 4, *light arrow*) and fatty streaks with more muscle than fat tissue (Gouttalier score 2, *dark arrow*) posteriorly. **d** The correspondent control to the patient in (**c**) evaluated as normal muscle tissue (Goutallier score 0–1)

**Fig. 2:**
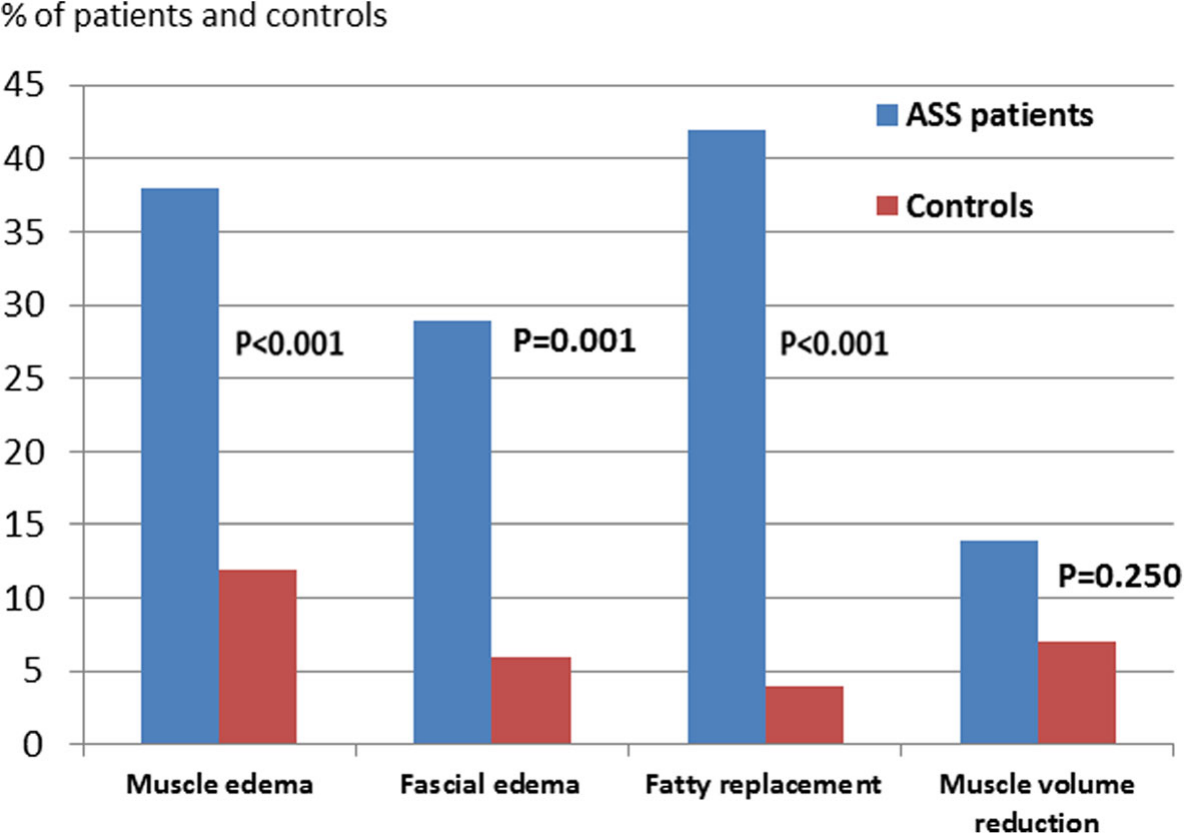
Proportions of ASS patients (*left: blue bars*) and controls (*right: red bars*) with muscle edema, fascial edema, fatty replacement and muscle volume reduction. *ASS* anti-synthetase syndrome (Color figure online)

**Fig. 3:**
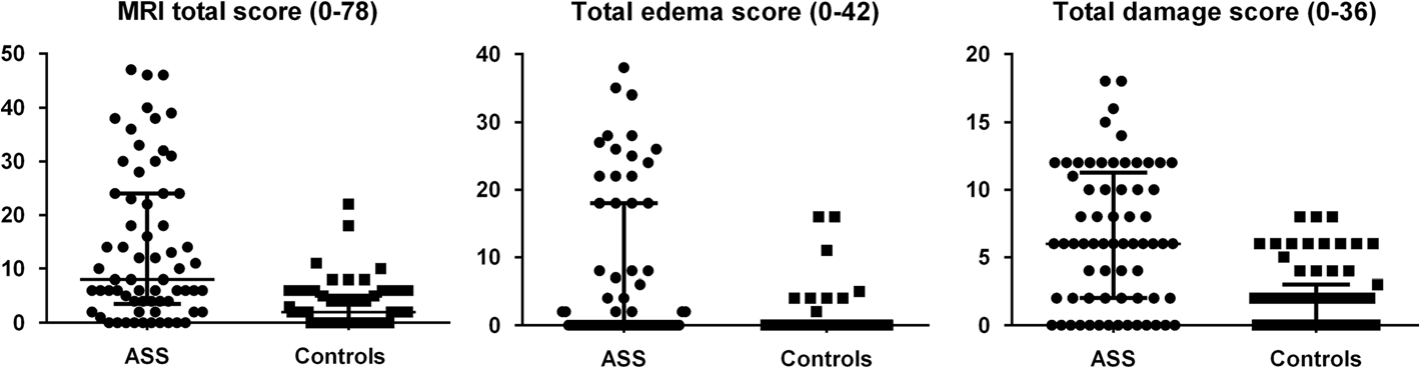
Median with interquartile range in ASS patients and controls for total MRI score (0–78), total edema score (0–42) and total damage score (0–36). All three scores showed significant differences between the two groups. *ASS* anti-synthetase syndrome, *MRI* magnetic resonance imaging

All controls (*N* = 67) and 66/68 ASS patients underwent MRI of the thigh muscles (Fig. 1). Two patients were unable to complete the examination due to claustrophobia. Defined MRI abnormalities were identified in 65% of the patients and 22% of the controls (*p* < 0.001). In 26% of the patients there were concomitant edema and muscle damage changes, compared with 1.5% of the controls (*p* < 0.001). Muscle edema was more frequent in patients (38%) than in controls (12%) (*p* < 0.001), as was fatty replacement (42% versus 4%; *p* < 0.001) (Fig. 2). The magnitude of changes differed between patients and controls; muscle edema score > 4 was present in 20 patients (*p* < 0.001), but only in three of the controls; and fatty replacement score ≥ 6 was seen in 40 patients and 11 controls (*p* < 0.001). Fascial edema was present in 29% of the ASS patients and in 6% of the controls (*p* < 0.001). Sixteen of the 19 ASS patients with fascial edema had known myositis. Muscle volume reduction was evident in nine patients (14%) and five controls (7%) (*p* < 0.250). In four patients and five controls the MRI changes were asymmetric. Total MRI score ranged from 0 to 47 in the ASS patients, compared to 0 to 22 in the controls (Fig. 3a). Total MRI score ≥ 10 was found in 31 patients and four controls (*p* < 0.001). Total edema score ranged from 0 to 38 and from 0 to 16 in the ASS patients and controls, respectively, and the range of total damage score was 0–18 in the patients and 0–8 in the controls (Fig. 3b, c). Compared with the controls, the ASS patients scored significantly higher in all three scores (total MRI, total edema and total damage) (Table 1 and Fig. 3).

**Table 1:** Muscle MRI scores and muscle parameters in ASS patients and controls

Muscle MRI scores	ASS (*N* = 66)	Controls (*N* = 67)	*p* value
Total edema score (0–42)			
Edema extent score (0–18)	3.2 (5.3)	0.4 (1.4)	<0.001
Edema signal intensity score (0–18)	3.1 (5.0)	0.5 (1.7)	<0.001
Fascial edema score (0–6)	1.3 (2.1)	0.1 (0.4)	<0.001
Total damage score (0–36)			
Fatty replacement score (0–30)	5.8 (4.3)	1.8 (2.4)	<0.001
Muscle volume reduction score (0–6)	0.6 (1.6)	0.1 (0.6)	<0.036
Total MRI score (activity + damage; 0–78)	14.1 (14.1)	3.0 (4.2)	<0.001
Other muscle parameters			
Median CK, U/L (range)	95 (24–1344)	98 (35–839)	<0.676
CK > 2 × ULN, *n*/*N* (%)	4/67 (6)	1/65 (2)	<0.366
Median MMT14 score (25th, 75th centile)	139 (133, 140)	140 (138, 140)	<0.001
Median MMT4 score (25th, 75th centile)	40 (36, 40)	40 (39, 40)	<0.004
Median FI2 score (25th, 75th centile)	211 (116, 301)	324 (281, 330)	<0.001

Values presented as mean (SD) if not stated otherwise

*ASS* anti-synthetase syndrome, *CK* creatinine kinase, *ULN* upper limit of normal, *MMT4* manual muscle test of four muscles, *MMT14* manual muscle test of 14 muscles, *FI2* functional index, *MRI* magnetic resonance imaging

### Distribution of MRI changes in the ASS patients

**Fig. 4:**
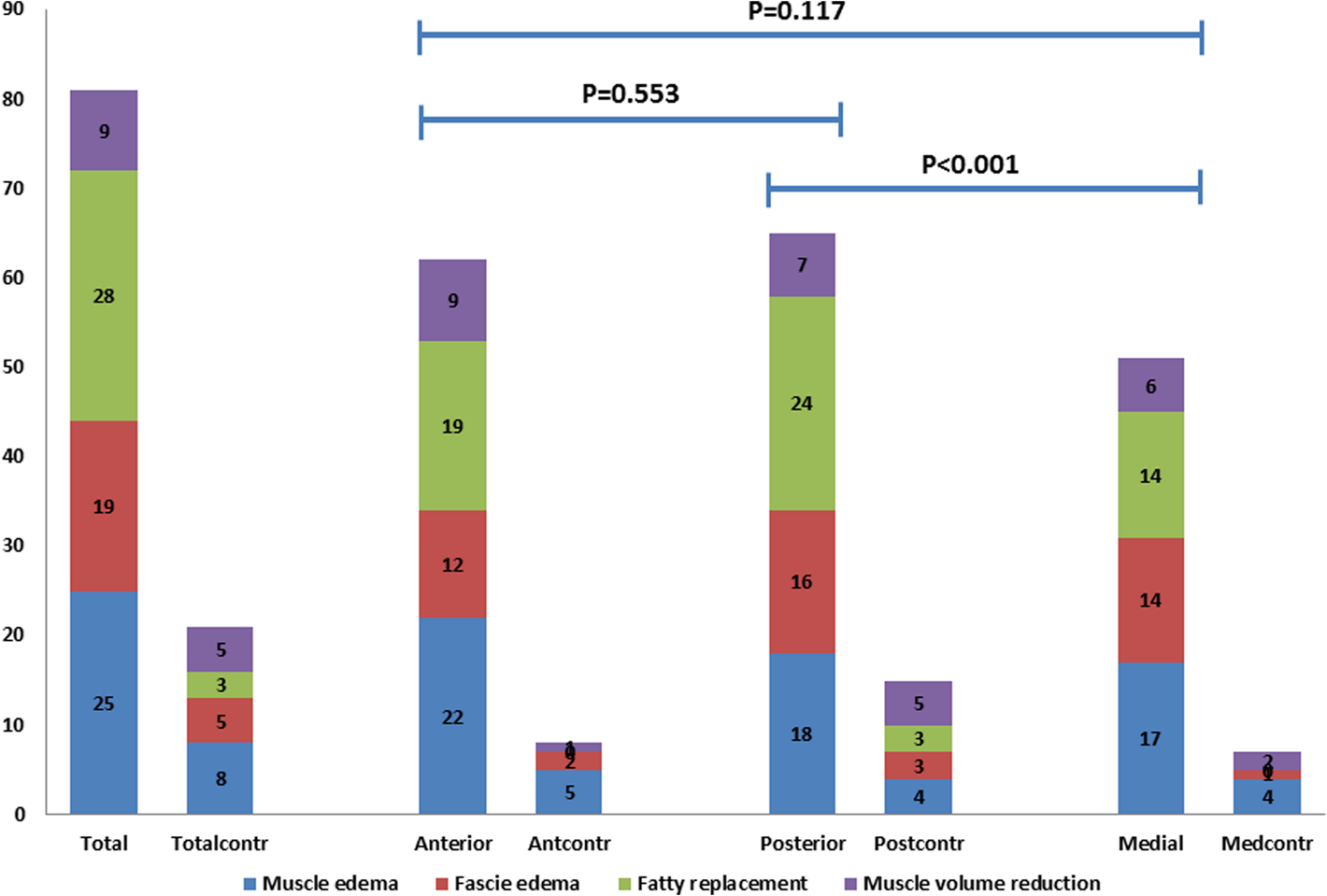
Accumulated numbers of MRI abnormalities identified in ASS patients (*N* = 66) and controls (*N* = 67). Because each person could have more than one type of MRI abnormality, the accumulated number of abnormalities exceeds the number of patients. The two panels to the left show the overall numbers (*total*) with muscle edema (*blue*), fascial edema (*red*), fatty replacement (*green*) and muscle volume reduction (*violet*), while the six panels to the right show the number of abnormalities in the anterior, posterior and medial compartments. *Contr* controls (Color figure online)

Muscle MRI changes were most common in the posterior compartment followed by the anterior and medial compartments (Fig. 4). Muscle edema was most prominent in the anterior compartment, evident in 1/3 of the patients, while fatty replacement was seen mostly posteriorly (in 36% of the patients) (Fig. 4). Fascial edema was distributed almost equal in the three compartments in about 21% of the patients.

### MRI muscle findings in different ASS subgroups

In ASS patients previously diagnosed with myositis, MRI changes were identified in 35/53 patients (66%); 10/53 (19%) had a total MRI score = 0. Mean (SD) total MRI scores were 15.9 (14.67) in the myositis group and 5.5 (3.57) in the no myositis group (*p* < 0.001) (Table 2). Unexpectedly, 5/13 of the ASS patients in the no myositis group had MRI changes consistent with muscle inflammation and/or muscle damage. Muscle strength and endurance did not differ between the groups (Table 2). When dividing the ASS patients into anti-Jo-1-positive and anti-Jo-1-negative groups, the results of MRI abnormalities, CK levels and muscle strength corresponded to those of the myositis/no myositis groups (Table 2).

**Table 2:** Demographics and MRI changes in subgroups of the ASS cohort

	Myositis diagnosis prior to inclusion			Anti-Jo-1 positivity			Myositis subtype		
	Yes (*N* = 54)	No (*N* = 14)	*p* value	Yes (*N* = 53)	No (*N* = 15)	*p* value	PM (*N* = 27)	DM^a^ (*N* = 25)	*p* value
Age at diagnosis (years)	47 (14.3)	49 (11.5)	n.s.	48 (13.9)	43 (12.8)	n.s.	50 (14.9)	43 (13.7)	n.s.
Disease duration (months)	**76 (6–362)**	**22 (7–230)**	**0.003**	**76 (6–362)**	**35 (7–230)**	**0.004**	70 (6–362)	83 (14–350)	n.s.
Jo1/non-Jo1, *n*	**49 / 5**	**4 / 10**	**<0.001**				25 / 2	23 / 2	n.s.
Ro-52 positivity, *n*/*N* (%)	38/54 (70)	7/14 (50)	n.s.	37/53 (70)	8/15 (53)	n.s.	17/27 (63)	17/25 (68)	n.s.
CK (U/L)	103 (38–1344)	71 (24–194)	n.s.	101 (38–1344)	70 (24–572)	n.s.	101 (43–437)	113 (38–1344)	n.s.
MMT14 score (0–140)	139 (134, 140)	136 (127, 140)	n.s.	139 (134, 140)	139.5 (131.5,140)	n.s.	139 (136, 140)	137 (134, 140)	n.s.
MMT4 score (0–40)	40 (36, 40)	38.5 (34, 40)	n.s.	39 (36, 40)	40 (35, 40)	n.s.	40 (37, 40)	39 (36, 40)	n.s.
FI2 score (0–330)	212 (125, 297)	194.5 (91,320)	n.s.	221.5 (129, 304)	185 (82, 301.5)	n.s.	224 (87.5, 318)	210 (129.5, 288)	n.s.
MRI changes									
Muscle edema score (0–36)	**7.49 (10.7)**	**0.62 (1.5)**	**<0.001**	**7.86 (10.7)**	**0.27 (1.0)**	**<0.001**	**0 (0–26)**	**4 (0–36)**	**0.039**
Total edema score (0–42)	**8.83 (12.0)**	**1.38 (2.5)**	**<0.001**	**9.29 (12.1)**	**0.80 (2.2)**	**<0.001**	6.0 (9.6)	11.6 (13.8)	n.s.
Total damage score (0–36)	**6.98 (5.4)**	**4.15 (2.5)**	**0.008**	**7.06 (5.5)**	**4.27 (2.7)**	**0.010**	7.0 (5.7)	7.1 (5.1)	n.s.
Total MRI score (0–78)	**15.87 (14.7)**	**5.54 (3.6)**	**<0.001**	**16.39 (14.7)**	**5.13 (3.8)**	**<0.001**	13.0 (13.0)	18.8 (15.6)	n.s.

Data presented as mean (SD), median (range) and median (25th, 75th centile) unless indicated otherwise

Data is presented as mean (SD) or median (range). For MMT14, MMT4 and FI2 data is presented with median (25th, 75th percentile)

^a^Except for two patients with amyopathic DM

*ASS* anti-synthetase syndrome, *MRI* magnetic resonance imaging, *PM* polymyositis, *DM* dermatomyositis, *n.s.* not significant, *CK* creatinine kinase, *MMT4* manual muscle test of four muscles, *MMT14* manual muscle test of 14 muscles, *FI2* functional index

Bold text refers to significant differences

The only MRI score item that differed between PM and DM subsets was muscle edema, where DM patients scored significantly higher than PM patients (*p* < 0.039; Table 2). Fascial edema was equally frequent in PM and DM (present in eight patients in each group).

Subgroup segregation by treatment showed that patients with ongoing steroid treatment (*N* = 57) had lower scores of muscle edema (*p* < 0.014) and, consequently, total edema score (*p* < 0.044) than patients without steroids (*N* = 9; data not shown). No correlation was found between steroid treatment, muscle volume reduction or total damage score.

The six patients without any immune suppressive treatment all had known myositis and normal CK values at study inclusion. These six patients had a median (range) total MRI score of 26.5 (0–39) with a median (range) total edema score of 23.5 (0–27) (data not shown).

### Plasma CK levels in ASS patients and controls

Plasma CK analyses were performed in 67 patients and 65 controls. No significant difference was found in median (range) CK levels between the groups (Table 1). Only 4/67 ASS patients and 1/65 controls had CK levels > 2 × ULN. Levels of CK correlated with total edema score in the ASS patients (*r* = 0.501, *p* < 0.01) but not in the controls (data not shown). Still, 23% of the ASS patients with a normal CK had total edema score > 18. All of these patients had established myositis. Retrospective CK data from the time of the ASS diagnosis was available in 55 patients. At diagnosis these patients had a median CK level of 308 (range 20–14,900), compared to 95 (range 24–1344) at study inclusion (*p* < 0.002). The CK values at diagnosis did not correlate with total edema score or total damage score.

### Muscle strength and endurance

The ASS group had significantly lower MMT14, MMT4 and FI2 scores than the matched controls (Table 1). In the ASS patients, significant correlations were seen between total damage score, MMT14 (*r* = –0.340, *p* < 0.01), MMT4 (*r* = –0.344, *p* < 0.01) and the FI2 test (*r* = –0.484, *p* < 0.01). None of the three muscle strength tests correlated with the total edema score.

### Factors associated with muscle activity and damage in ASS

In multiple linear regression analyses, the total edema score was associated with pathological CK levels (*p* < 0.001) and with anti-Ro52 and anti-Jo1 positivity (*p* < 0.016 and *p* < 0.036, respectively). No association was seen with age, gender, disease duration, CK levels at diagnosis or ongoing treatment. The total damage score in ASS was associated with age (*p* < 0.002) and disease duration (*p* < 0.015).

## Discussion

Data on muscle MRI findings and muscular outcome in ASS are limited. Here we showed that MRI findings compatible with either muscle damage (i.e. fatty replacement and/or reduction in muscle volume) or active myositis (edema in muscle and/or fascia) were present in 65% of the patients in an ASS cohort with a median 6 years of disease duration. Highly significant differences between patients and healthy controls were seen in all MRI muscle parameters, muscle strength and endurance.

Muscle MRI has been performed in a number of different muscle conditions, using semiquantitative and quantitative methods [30–34]. Taken together, these data indicate that some considerations should be made when evaluating muscle MRI in myositis. First, muscle MRI changes do not have to represent myositis. In this study, we also identified edema, fatty replacement and volume reduction in the healthy controls. However, the MRI changes in healthy controls were much less pronounced than in the patients; only three controls had a muscle edema score > 4 and only one control had concomitant signs of muscle edema and damage. Two controls had edema evaluated as post-traumatic changes, a well-known cause of MRI muscle edema [35]. The four controls with total MRI score ≥ 10 were all over 70 years of age. This is relevant because the total MRI score in the controls correlated with age at study inclusion. Second, muscle MRI changes can also be influenced by gender, especially muscle volume. Because no definitions of normal muscle volumes exist, muscle atrophy is difficult to assess. By using healthy controls matched for gender, and a method which would underestimate rather than overestimate muscle atrophy, we tried to reduce this problem of interpretation. However, we still believe that the data on muscle volume reduction must be interpreted with some caution. Interestingly, we discovered a high frequency of fatty streaks (Gouttalier score = 1) in muscle compartments both in patients (33%) and in controls (40%). Not surprisingly, the frequency of fatty streaks (and the fatty replacement score) also correlated with age at study inclusion in both groups, possibly indicating that a “minor extent” of fatty replacement could be a natural finding in muscle anatomy.

The current study did not reveal any specific ASS-related MRI pattern equivalent to the recently described ASS-related muscle histology pattern with necrotic myofibers in the perimysium and inflammation in adjacent connective tissue [21, 22]. However, some potentially interesting observations were made. First, we found that 16 patients (eight PM and eight DM) were described with fascial edema and 13/16 of these had concurrent muscle edema. Previously, fascial edema has mostly been described in DM and found to correlate with histopathological findings [13, 36]. We speculate that the observed MRI pattern with concurrent edema in muscle and fascia is a feature of active ASS, regardless of PM/DM subset, but this needs to be evaluated in further studies. Second, we found a trend toward partition of the MRI findings, with predominance of fatty replacement in the posterior compartment and muscle edema in the anterior compartment. Because this study had a cross-sectional design, we do not know whether the more pronounced damage in the posterior musculature was a consequence of relatively early and/or intense inflammation in this compartment. Interestingly, fatty replacement (Gouttalier score ≥ 2) was also present in a small number of the controls (*N* = 3), all in the posterior part of the thighs, so these findings need to be further investigated. A similar MRI pattern, with edema anteriorly and fatty replacement posteriorly, has recently been described in PM patients, including patients with immune-mediated necrotizing myopathy (IMNM) [14, 30].

The total edema score correlated with CK levels. This is consistent with recent data from Italy where muscle edema and CK correlated in 51 IIM patients [37]. However, in contrast to other studies [37, 38], there was no correlation between the three muscle tests evaluated and the total edema score. Instead, we found that muscle strength correlated with the total damage score. Hence, it is possible that the current study design, with cross-sectional evaluation of patients with established disease, is most suitable for MRI assessment of chronic muscle changes. It should, however, be noted that 26% of the ASS patients with visible muscle damage had concurrent signs of activity (edema), and that 23% of the ASS patients, all with normal CK values, had a total edema score ≥ 43% of maximum. Because muscle edema was also seen in 12% of the controls (all but one with normal CK values), one could discuss the clinical importance of this finding. It should, however, be noted that the extent and intensity of edema in the controls was far less than in the patients. In fact, the 90th centile of total edema score in the controls was only 4, compared to 26 in the patients. In addition, the fact that the ASS patients with no previous myositis diagnosis (*N* = 13) had higher MRI scores than their correspondent healthy control (*p* < 0.02) emphasizes the importance of muscle MRI to evaluate the possibility of late-onset/subclinical myositis in ASS.

It has earlier been described that anti-Ro52 is associated with poor outcome in ASS-related ILD [39, 40], and Marie et al. [41] have also reported anti-Ro52 as a prognostic factor for the muscle component in the syndrome. This was also seen in the current study, independently of anti-Jo1 status (*p* < 0.05). Hence, anti-Ro52 seems to be a possible prognostic factor for both the lung and muscle components in ASS. Furthermore, subgroup analyses showed an association between anti-Jo1 positivity and total edema score in the patients with earlier diagnosed myositis. These findings should be evaluated carefully, however, since the anti-Jo-1-negative myositis group was small (*N* = 5).

The study has some potential limitations. First, the cross-sectional study design limits evaluation of potential predictive muscle MRI variables and the progression of these. Second, muscle endurance was also tested in muscle groups other than thigh muscles; it is therefore possible that whole-body MRI could correlate better with the FI2 test. Third, we did not specifically investigate possible associations between different kinds of treatment, MRI findings and muscular functions in the ASS patients.

## Conclusions

This comparative study showed that ASS patients, after a median disease duration of nearly 6 years, had significantly more muscle edema, fascial edema, fatty replacement and muscle volume reduction than age and gender-matched controls. The study emphasizes MRI as an independent tool in the assessment of ASS-related myositis; particularly in cases with normal CK. Prospective studies are needed to determine the predictive values of MRI activity signs and the rules governing progression of MRI damage items.

## Additional file

Additional file 1: Figure S1. showing the distribution of myositis subsets in ASS patients with and without anti-Jo1 antibodies. *Blue* polymyositis (*PM*), *light blue* dermatomyositis (*DM*), *red* clinical amyopathic dermatomyositis (*CADM*) and *yellow* no myositis. (TIF 31 kb)

## Abbreviations

aaRS: Amino-acyl t-RNA synthetase
ASS: Anti-synthetase syndrome
CK: Creatinine kinase
DM: Dermatomyositis
FI2: Functional index 2
IIM: Idiopathic inflammatory myopathies
ILD: Interstitial lung disease
IMNM: Immune-mediated necrotizing myopathy
MMT: Manual muscle test
MRI: Magnetic resonance imaging
PM: Polymyositis
SPSS: Statistical package for the social sciences
STIR: Short T1 inversion recovery
ULN: Upper limit of normal
