# Supplementary figures and images for: Comparative analyses of muscle MRI and muscular function in anti-synthetase syndrome patients and matched controls: a cross-sectional study
Source: Arthritis Res Ther. 2017 Jan 25;19:17. doi: 10.1186/s13075-017-1219-y (PMC5264447; doi:10.1186/s13075-017-1219-y)

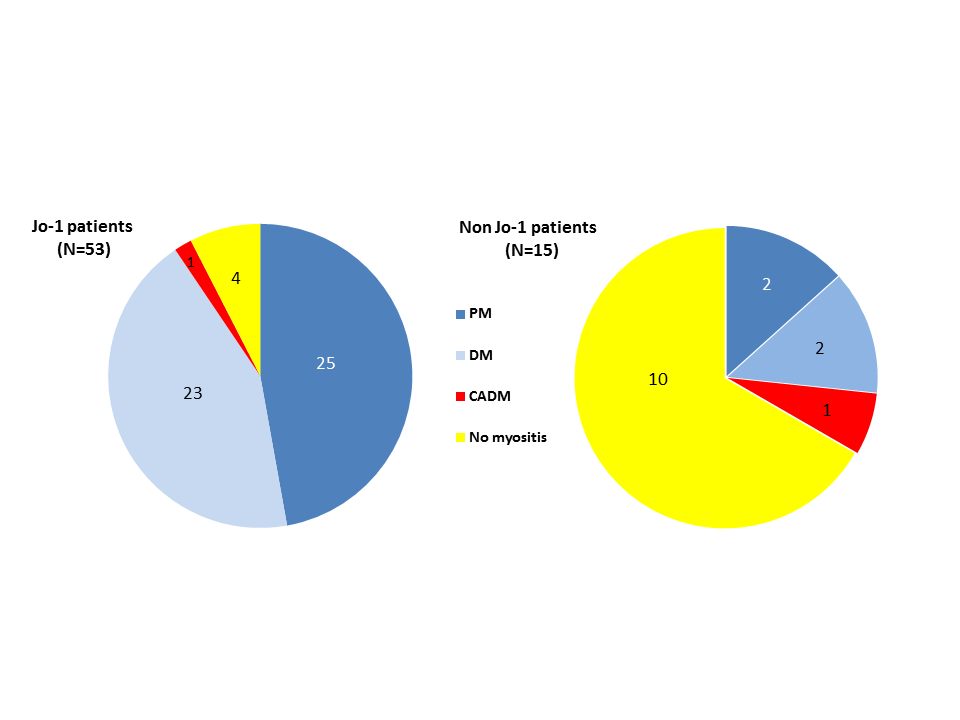

Supplement: Additional file 1: Figure S1. — showing the distribution of myositis subsets in ASS patients with and without anti-Jo1 antibodies. Blue polymyositis (PM), light blue dermatomyositis (DM), red clinical amyopathic dermatomyositis (CADM) and yellow no myositis. (TIF 31 kb) [file 13075_2017_1219_MOESM1_ESM.tif]
